# Fermitins, the Orthologs of Mammalian Kindlins, Regulate the Development of a Functional Cardiac Syncytium in *Drosophila melanogaster*

**DOI:** 10.1371/journal.pone.0062958

**Published:** 2013-05-15

**Authors:** James H. Catterson, Margarete M. S. Heck, Paul S. Hartley

**Affiliations:** The University of Edinburgh/British Heart Foundation Centre for Cardiovascular Science, The University of Edinburgh, Edinburgh, Midlothian, United Kingdom; University of Otago, New Zealand

## Abstract

The vertebrate Kindlins are an evolutionarily conserved family of proteins critical for integrin signalling and cell adhesion. Kindlin-2 (KIND2) is associated with intercalated discs in mice, suggesting a role in cardiac syncytium development; however, deficiency of *Kind2* leads to embryonic lethality. Morpholino knock-down of *Kind2* in zebrafish has a pleiotropic effect on development that includes the heart. It therefore remains unclear whether cardiomyocyte *Kind2* expression is required for cardiomyocyte junction formation and the development of normal cardiac function. To address this question, the expression of *Fermitin 1* and *Fermitin 2 (Fit1, Fit2)*, the two *Drosophila* orthologs of *Kind2*, was silenced in *Drosophila* cardiomyocytes. Heart development was assessed in adult flies by immunological methods and videomicroscopy. Silencing both *Fit1* and *Fit2* led to a severe cardiomyopathy characterised by the failure of cardiomyocytes to develop as a functional syncytium and loss of synchrony between cardiomyocytes. A null allele of *Fit1* was generated but this had no impact on the heart. Similarly, the silencing of *Fit2* failed to affect heart function. In contrast, the silencing of *Fit2* in the cardiomyocytes of *Fit1* null flies disrupted syncytium development, leading to severe cardiomyopathy. The data definitively demonstrate a role for *Fermitins* in the development of a functional cardiac syncytium in *Drosophila*. The findings also show that the *Fermitins* can functionally compensate for each other in order to control syncytium development. These findings support the concept that abnormalities in cardiomyocyte KIND2 expression or function may contribute to cardiomyopathies in humans.

## Introduction

The mammalian Kindlins are a family of evolutionarily conserved proteins that mediate cell-cell and cell-matrix adhesion by regulating integrin function [1]–[3]. Findings from several animal models suggest that the formation of junctions between neighbouring cardiomyocytes (intercalated discs) may require integrins and integrin binding proteins [4]–[8]. It is important to understand the role of these proteins because mutations and polymorphisms that affect intercalated disc formation may be associated with the development of cardiomyopathies in humans [6], [9], [10].

Murine KIND2 protein is detected at intercalated discs, however genetic ablation of *Kind2* in mice causes early embryonic lethality at the peri-implantation stage, before cardiogenesis [11], [12]. In zebrafish, the global knock-down of *Kind2* causes cardiac hyperplasia, disrupts intercalated disc formation and significantly reduces cardiac contractility [11]. These findings link *Kind2* with the development of a functional cardiac syncytium; however a cardiomyocyte-specific test of this hypothesis is yet to be conducted.

**Figure 1:**
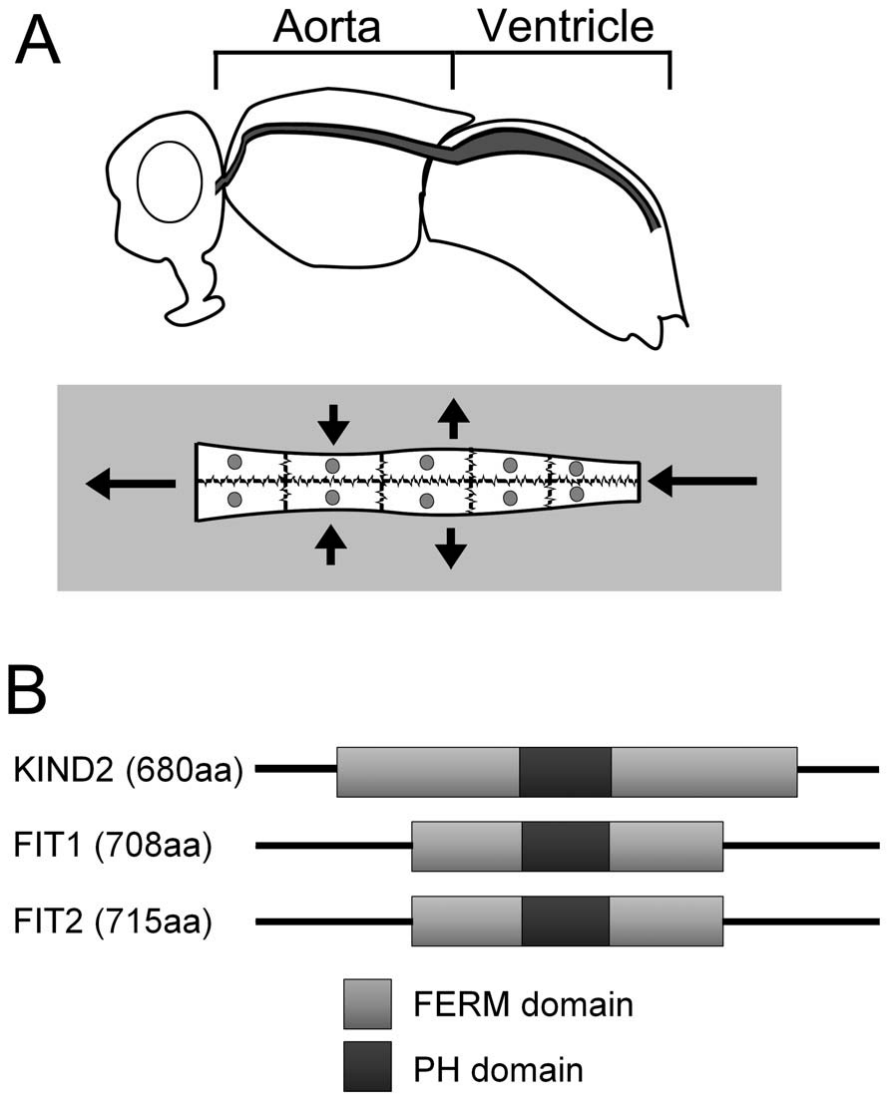
Overview of the adult *Drosophila* heart and *Fermitins*. (**A**) The schematic illustrates the position of the *Drosophila* heart (shaded grey) in the adult fly. The contractile portion of the heart (the ventricle) is within the abdomen and is connected directly to a non-contractile aorta which directs haemolymph (the insect equivalent of blood) from the abdomen, through the thorax and onwards to the head. The second schematic illustrates the ventricle, which consists of paired cardiomyocytes that function together as a single contractile syncytium (small arrows). Long arrows show the direction of circulation; however the heart can contract in different directions, so as to direct haemolymph to the fly's anterior or posterior. (**B**) The integrin binding FERM domain of human Kind2 is present in both Fit1 and Fit2.

*Drosophila melanogaster* is an important model for the identification and study of genes that have relevance to mammalian cardiovascular development and function [13]–[17]. The abdominal heart in *Drosophila* consists of individual cardiomyocytes that form a contractile syncytium and pump haemolymph from the abdomen, through the thorax towards the head (see schematic in Figure 1A).

*Drosophila* expresses two orthologs of human KIND2, *Fermitin1* (*Fit1*) and *Fermitin2* (*Fit2*) that contain a highly conserved FERM (4.1, Ezrin, Radixin, Moesin) domain which is required for binding to the cytoplasmic tail of β-integrins [18] (see Fig. 1B and Fig. S1). Both *Fit1* and *Fit2* message and protein are detected in adult heart tissue, consistent with a role in cardiac function [19], [20].

To explore the role of Fermitins in the *Drosophila* heart we silenced their expression in cardiomyocytes and analysed the function of the adult heart. It was found that cardiomyocyte expression of *Fermitins* is critical for the development of a functional syncytium in *Drosophila* and that the genes can functionally compensate for each other. The findings support the hypothesis that disruption of cardiomyocyte *KIND2* may lead to abnormal intercalated discs and cardiomyopathies in humans.

## Methods

### *Drosophila* stocks and fly husbandry

Flies were raised on standard medium at 25°C on a 12/12 hr light/dark schedule. Flies used in this paper were *w^1118^*, *Fit1^KG05576^*, and *Fit2^EY08530^* were obtained from the Bloomington stock centre. *HandC-GAL4* 4.2 [21] (*Hand-Gal4*) flies were kindly provided by A. Paululat. *HandC-GAL4; tub-GAL80^ts^*, *UAS-Fit1^VDRC^; UAS-Fit2^VDRC^*, and *HandC-GAL4; Fit1^Δ161^ UAS-Fit2^VDRC^* flies were generated following standard crosses. Vienna *Drosophila* RNAi Centre (VDRC) RNAi lines used – Fit1 (#46494), Fit2 (#37010), and Mys (#29619). Transgenic RNAi Project (TRiP) RNAi lines used – Fit1 (#25966).

### Genomic PCR and RT-PCR

For genomic DNA, whole flies were homogenised in lysis buffer (1M TrisHCL pH 7.5, 500 mM EDTA, pH 8.0, 4M NaCl, 10% SDS)(Sigma, Poole, Dorset, UK) using Kontes pellet pestle (Fisher Scientific, Loughborough, UK) and incubated at 65°C for 30 mins. LiCl/KAc solution (6M LiCl, 5M KAc)(Sigma, Poole, Dorset, UK) was then added, mixed, and incubated on ice for 10 mins. Samples were centrifuged for 15 mins at 12,000 rpm at room temperature. Supernatant was transferred to fresh 1.5 mL centrifuge tubes and isopropanol (VWR, Lutterworth, Leicestershire, UK) added to precipitate DNA. Samples were then centrifuged for 20 mins at 12,000 rpm at room temperature. Pellet was washed with cold 70% EtOH (VWR, Lutterworth, Leicestershire, UK) and centrifuged for 10 mins at 12,000 rpm at room temperature. DNA pellets were left to air dry for 1-hour then resuspended in Tris-EDTA (10 mM Tris, 1 mM EDTA)(Sigma, Poole, Dorset, UK) overnight at 4°C. For fly heart RT-PCR, total RNA was extracted from 20 dissected hearts of 1-wk-old adult females using TRIzol (Invitrogen, Paisley, UK) phenol-chloroform extraction method with isopropanol precipitation. RNA was resuspended in nuclease-free DEPC-water and treated with RQ1 RNase-free DNase (Promega, Southampton, UK), then cDNA was generated with M-MLV Reverse Transcriptase (Promega, Southampton, UK) using oligo-dT primer and RNasin Plus RNase Inhibitor (Promega, Southampton, UK). PCR was performed using relevant primers, dNTP Mix (Promega, Southampton, UK), and GoTaq DNA Polymerase in 5X Green GoTaq Reaction Buffer (Promega, Southampton, UK). The primer sets were as follows: for RP49, 5′-GACAATCTCCTTGCGCTTCT-3′ and 5′-CCAGTCGGATCGATATGCTAA-3′; for Fit1, 5′-AACAGTGAGGTCTGGGTGAGAT-3′ and 5′-AACAGCTCCTCCTTCTTGTGTC-3′; for Fit2, 5′-ACGGTATCAACAGTGAGGTGTG-3′ and 5′-GATGCCCGTCGAACTTAATG-3′ (Eurofins MWG Operon, Acton, London, UK). PCR samples were then analysed by gel electrophoresis and images taken with Uvitec Gel Doc system (Uvitec, Cambridge, UK).

### Analysis of cardiac synchrony and generation of cardiac raster plots

A video of the beating heart was opened in ImageJ [22], converted to greyscale and a movement movie processed by subtracting a frame's pixel intensity from that of the preceding frame using the ‘Image Calculator’ function. The mean pixel intensity of two, non-overlapping regions (the upper left quartile and lower right quartile) of each frame in the movement movie was then measured using the ‘Measure stack’ function. Regression analysis was then performed on the mean pixel intensity data obtained from the two regions. When the two regions are in perfect synchrony, *R^2^* = 1; when regions are not in synchrony, *R^2^* approaches zero. The mean (±SEM) *R^2^* value of 7 flies for each genotype is presented. Cardiac raster plots were generated by concatamerising the image stills from the movement movies in ImageJ so that there were 12 consecutive images per row over 30 columns. The resulting raster plot corresponds to 10.8 seconds of cardiac motion. Under normal conditions a rhythmically occurring event produces a geometric pattern in a raster plot, whereas loss of rhythmicity leads to a more random pattern. This methodology of illustrating a physiological rhythm is based on the analysis of biological rhythm data in the circadian biology field.

### Fluorescent labelling and imaging of adult heart structures

Fluorescent labelling of *Drosophila* heart structures was performed as described [23]. In brief, semi-intact heart preparations of four flies of each genotype were fixed in 4% formaldehyde (Fisher Scientific, Loughborough, UK) in PBS for 20 mins then 3×10 min washes with PBS-Triton-X (PBST)(Sigma, Poole, Dorset, UK). Hearts were then incubated with primary antibodies and/or stained in PBST overnight at 4°C with constant agitation. Hearts were washed 3×10 mins in PBST, incubated with secondary antibodies for 1-hour at room temp, washed 3×10 mins in PBST plus final wash in PBS alone. Hearts were then mounted in 50% glycerol (Sigma, Poole, Dorset, UK) in PBS and visualised using a Zeiss Axioskop 2 mot plus fluorescent microscope and images were taken using Openlab imaging software. Anti-βPS-integrin (1∶10 of hybridoma supernatant (100 µg/ml), Developmental Studies Hybridoma Bank) was used to stain cell junctions, AlexaFluor 546-conjugated phalloidin (13.2 nM) to stain F-actin (Invitrogen, Paisley, UK), and DAPI (1 ng/ml) to stain nuclei (Sigma, Poole, Dorset, UK). The hybridoma developed by [24] was obtained from the Developmental Studies Hybridoma Bank developed under the auspices of the NICHD and maintained by The University of Iowa, Department of Biology, Iowa City, IA 52242.

### Measurement of distance between neighbouring cardiomyocytes

ImageJ (http://rsbweb.nih.gov/ij/) was used to measure the distance between neighbouring cardiomyocytes. In ImageJ, the ‘straight line’ drawing tool was selected and a line was drawn between neighbouring cardiomyocytes. The scale was calibrated (Analyse>Set Scale) to measure in microns, and distances were measured using the ‘Measure’ function (Analyse>Measure, or CTRL+M) in ImageJ. Four measurements were taken for each fly heart, and up to 7 flies were used for each genotype – resulting in 12–28 measurements from 4–7 independent flies for each genotype. Data was then grouped for each genotype and mean +/− SEM was calculated.

### Heart preparation and functional assays

It is necessary to surgically expose the fly heart for detailed analysis of cardiac contractions – making them accessible for video microscopy [25]. We used the previously published heart assay and analysis methodology [26], and used between 6 and 11 female flies, aged 5–7 days old for all analyses. In brief, the ventral abdomen was dissected in artificial haemolymph (108 mM NaCl, 5 mM KCl, 2 mM CaCl_2_, 8 mM MgCl_2_, 1 mM NaH_2_PO_4_, 4 mM NaHCO_3_, 5 mM HEPES, 10 mM sucrose, 5 mM trehalose) to expose the fly's heart, which lines the dorsal cuticle, and digital movies of the heart over body segments A2 and A3 were taken (video acquisition by Pinnacle Software (Pinnacle Systems, Iver Heath, UK)). Video footage was analysed using a MatLab-based image analysis program, M-modes were generated and cardiac parameters including heart rate, arrhythmias, heart periods, diastolic and systolic diameters, and fractional shortening were quantified [27]. Data was then grouped for each genotype and mean +/− SEM was calculated.

### Generation of Fit1^Δ161^ deletion line

The P-element inserted in *Fit1^KG05576^* was mobilized by following standard crosses. Mobilization events were screened by PCR using different primer combinations in the genomic DNA flanking the P-element – *Fit1^Δ161^* (5′- CATCCGTTCCGATAAGTTCG-3′ and 5′- CCAACAGCTCCTCCTTCTTG-3′) resulted from the imprecise excision of the P-element and creates an out-of-frame deletion which removes 1685 bp (629 bp from 5′ UTR region and 1055 bp from coding sequence)(deletion corresponds to nucleotides 67–1752) and retains a 12 bp fragment (CATGATGAAATA) from the start of the 11,467 bp P-element.

### Statistics

One-way analysis of variance (ANOVA) followed by Tukey's post-hoc test was used to identify differences between three or more means derived from uneven sample sizes. The student's unpaired t-test was used to identify significant differences between two means of uneven sample size. A statistical difference of P<0.01 was regarded as significant.

## Results

### *Fermitins* regulate cardiac syncytium development

**Figure 2:**
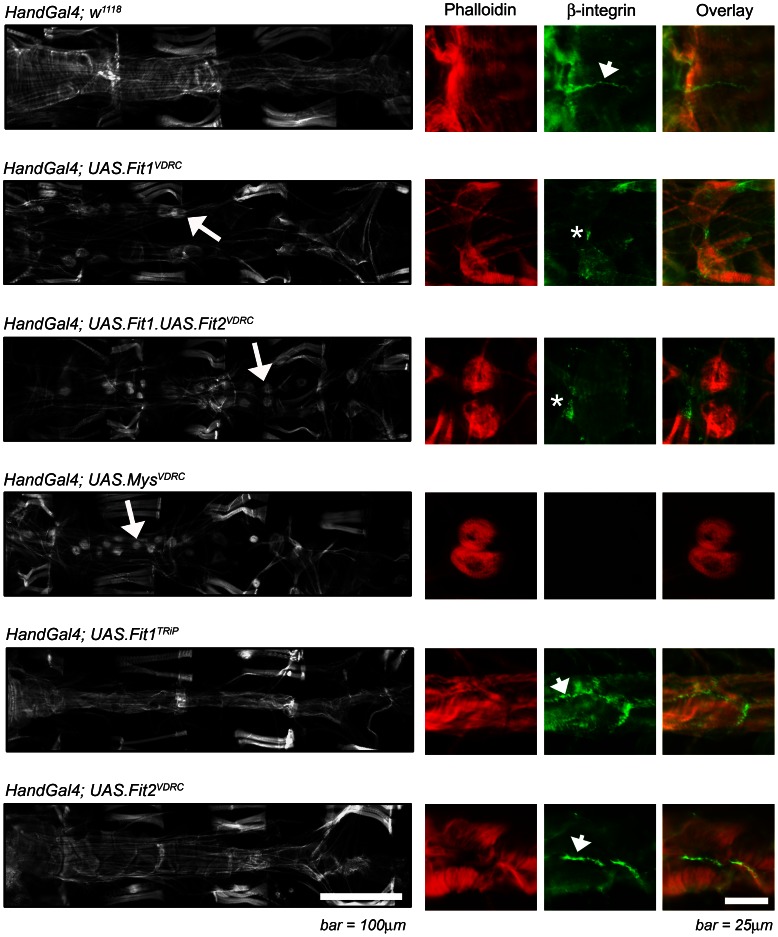
*Fermitins* regulate coupling between cardiomyocytes. The monochrome micrographs show the adult *Drosophila* heart (segments A2 to A5) stained with phalloidin, oriented with the anterior region to the left. Genes were silenced using the *Hand-Gal4* enhancer which drives expression in cardiomyocytes (as well as pericardial nephrocytes and enterocytes of the gut) and abnormal cardiomyocyte/heart morphology is highlighted by the arrows. The colour panels show higher magnification micrographs of at least two cardiomyocytes stained with phalloidin (red) and antibodies to the *Drosophila* β-integrin, *myospheroid* (green); arrowheads indicate normal integrin staining between cardiomyocytes; asterisks indicate an abnormal staining pattern associated with loss of cardiomyocyte junction integrity. A wild type phenotype (*Hand-Gal4; w^1118^*), is characterised by contiguous cardiomyocytes and β-integrin staining between cardiomyocytes.

To assess the effect of *Fermitin* knock-down on adult heart morphology, the cardiac *Hand-GAL4* driver was used to disrupt gene expression. In control flies the cardiomyocytes display a characteristic spiral myofibrillar arrangement that can be visualised by phalloidin staining, with β-integrin staining junctions between adjacent cardiomyocytes (Figure 2, *Hand-Gal4; w^1118^*). Silencing *Fit1* using the RNAi line from the Vienna *Drosophila* RNAi centre (VDRC [28]) led to loss of the cardiac syncytium and loss of contiguous β-integrin staining between adjacent cardiomyocytes)(Fig. 2, *Hand-Gal4; UAS.Fit1^VDRC^*). When both *Fit1* and *Fit2* were silenced, a more severe phenotype developed (Fig. 2, *Hand-Gal4; UAS.Fit1/Fit2^VDRC^*), with cardiomyocytes exhibiting a ‘rounded-up’ phenotype, comparable to that resulting from the silencing of the β-integrin *myospheroid* in cardiomyocytes (Fig. 2, *Hand-Gal4; UAS.Mys^VDRC^*). In contrast, when *Fit1* was silenced using the Harvard TRiP (Transgenic RNAi Project) RNAi line (Fig. 2, *Hand-Gal4; UAS.Fit1^TRiP^*), the cardiac syncytium developed normally and the cardiomyocytes exhibited a cytoskeletal morphology similar to that seen in controls. Similarly, when *Fit2* was silenced on its own, the cardiac syncytium developed normally (Fig. 2, *Hand-Gal4; UAS.Fit2^VDRC^*). The discrepancy between the phenotype caused by the *Fit1^VDRC^* and *Fit1^TRiP^* RNAi lines can be explained by the VDRC line targeting both *Fit1* and *Fit2* message (see Fig. S2), whereas the TRiP line targets only *Fit1*.

**Figure 3:**
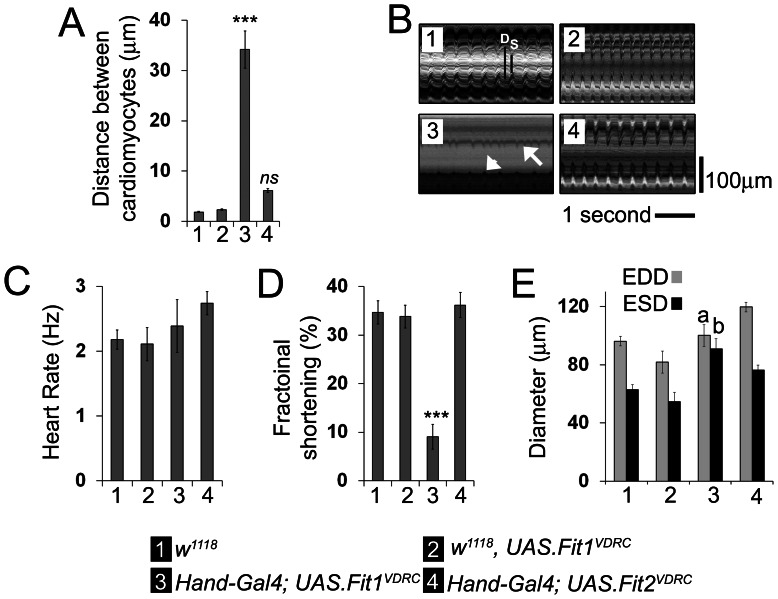
Silencing *Fermitin* expression prevents cardiomyocyte coupling and disrupts heart contractility. (**A**) The distance between nearest neighbouring cardiomyocytes was measured for four different genotypes. *n* = 16–28 measurements from four independent flies for each genotype. ***P<0.001, *ns* = not significantly different from any other genotype. (**B**) Representative M-modes illustrating heart contractions in the different genotypes. The white ‘D’ and ‘S’, and the black bars in the first M-mode denote the heart at diastole and systole. The arrow and arrowhead highlight reduced contractility and loss of synchrony between different regions of the heart. (**C**) Mean (±SEM) heart rate and (**D**) Fractional shortening (Diastolic diameter_MAX_ – Systolic diameter_MAX_). (**E**) Quantification of end diastolic and systolic diameter (EDD and ESD, respectively). ***P<0.001, *n* = 6–7 flies per genotype. a = not significantly different from controls' diastolic diameter; b = significantly different (P<0.01) from controls systolic diameter. Legend applies to A–E.

To confirm the effect of Fermitin knock-down on the heart the distance between neighbouring cardiomyocytes was quantified (Fig. 3A). In control flies and flies where no effect of an RNAi was observed (*w^1118^* flies crossed with the *UAS.Fit1^VDRC^* line in the absence of the *Hand-Gal4* driver), the distance between adjacent cardiomyocytes were never more than 3 µm. In contrast, the distance between cardiomyocytes when *Fit1* was silenced using the VDRC line, was ten-fold greater (34.2±3.7 µm, (P<0.001)). When *Fit2* was silenced there appeared to be a small effect on distances between cardiomyocytes, however this did not reach statistical significance.

### Silencing *Fermitin* expression disrupts heart contractility

The cardiac syncytium in vertebrates is a critical determinant of the heart's contractility. It was therefore reasoned that loss of the syncytium caused by *Fermitin* knock-down in *Drosophila* would dramatically impact cardiac function and this hypothesis was tested in adult flies using videomicroscopy. It was found that the cardiomyocytes of *Fit1^VDRC^* silenced flies (in which both *Fit1* & *Fit2* are silenced) beat at the same rate as controls (*w^1118^* and *w^1118^* crossed with *Fit1^VDRC^*) and flies in which *Fit2* had been knocked down (Figure 3C, P>0.05). However the distance the heart moved between diastole and systole (fractional shortening) was greatly reduced in *Fit1^VDRC^* silenced flies, compared to all other genotypes (see the M-mode in Figure 3B and the quantified data in Figure 3D; P<0.001). Consistent with this finding, the end systolic diameter was greater in the *Fit1^VDRC^* silenced flies than the other genotypes (Figure 3E, P<0.01); whereas diastolic diameter was similar to the controls (P>0.05). Examples of the beating hearts from the different genotypes and the disruption caused by the *UAS.Fit1^VDRC^* line can be seen in videos S1 to S4.

### Synchrony between cardiomyocytes is reduced when *Fermitin* expression is silenced

**Figure 4:**
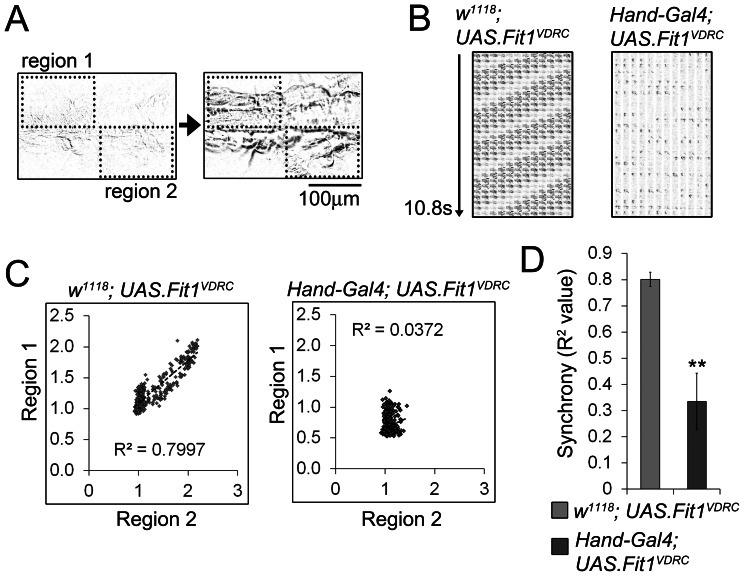
Synchrony between cardiomyocytes is reduced when *Fermitin* expression is silenced. Videomicroscopy of the contracting heart from control (*w^1118^, UAS.Fit1^VDRC^*) or *Fermitin*-silenced flies (*Hand-Gal4; UAS.Fit1^VDRC^*) was analysed for movement to ascertain the degree of synchronicity between the different (depicted) regions of the *Drosophila* heart. (**A**) Frames from a movement movie of the beating heart where dark pixels indicate movement relative to the previous frame. Pixel intensity in two non-overlapping regions is measured over 10 s and regression analysis performed to quantify the degree of synchrony between the two regions. (**B**) Concatamerised frames from the movement movie were used to develop cardiac actograms which illustrate the regular, synchronous movement of the heart in a control fly (*w^1118^, UAS.Fit1^VDRC^*), and asynchronous movements in the heart of a *Fermitin* silenced fly (*Hand-Gal4; UAS.Fit1^VDRC^*). The 360 frames run left to right and top to bottom, the entire sequence being 10.8 s long. (**C**) Representative XY scatter plots of pixel intensity in two regions of a 300 frame, 10 s movement movie, note the lower R^2^ value for the *Fermitin* silenced fly. (**D**) Synchrony (mean R^2^ values (±SEM)) for control (*w^1118^, UAS.Fit1^VDRC^*) and Fermitin silenced flies (*Hand-Gal4; UAS.Fit1^VDRC^*). *n* = 7 flies for each genotype, **P<0.01.

It was clear from the functional studies that cardiomyocytes in the *Fit1^VDRC^* silenced flies were capable of beating at a frequency similar to that of controls, but that fractional shortening was dramatically reduced. It was also clear that cardiomyocytes were no longer in close contact with each other and it was therefore reasoned that synchrony between cardiomyocytes had been lost. This loss of synchrony is a novel phenotype in *Drosophila* and was quantified by analysing movement movies from videomicroscopy footage that depict the motion of the heart during the cardiac cycle. To provide a 2-dimensional example of heart movement we explored the use of ‘actograms’, which are used to depict cyclical events over a given time period, especially in the field of biological rhythm research. In these images, a time-series of a measured or recorded parameter (e.g. behaviour, movement or gene expression) is presented as a raster plot (i.e. plotted left to right, top to bottom). A rhythmic system will produce a geometric pattern in a raster plot, whereas a random pattern is produced when a system is asynchronous. To illustrate the phenotype of the abnormal hearts in this way we concatamerised stills from the movement movies to generate raster plots of 10.8 seconds of heart activity and compared the plots from control flies (*w^1118^, UAS.Fit1^VDRC^*) with those from the *Fermitin* silenced flies (*Hand-Gal4; UAS.Fit1^VDRC^*, Fig. 4A, B). Using this method it was easy to identify the loss of rhythmicity as a transition from the geometric pattern of the control heart to the disordered plot from the *Fermitin* silenced fly. In order to quantify the degree of synchrony in different areas of the heart, two independent regions of the movement movie were compared to see if changes in pixel intensity (i.e. a measurement of cardiac motion) were in synchrony or not. Using simple regression analysis of two independent regions of the contracting heart in the movies, it was confirmed that synchrony was significantly reduced in *Hand-Gal4; UAS.Fit1^VDRC^* flies compared to control flies (*w^1118^, UAS.Fit1^VDRC^*; mean *R^2^* values, of 0.34±0.11 and 0.81±0.03, respectively (P<0.001, Figure 4C & D). Examples of the beating hearts and movement movies from the different genotypes can be seen in videos S5 to S8.

### *Fit1* is dispensable for syncytium development

**Figure 5:**
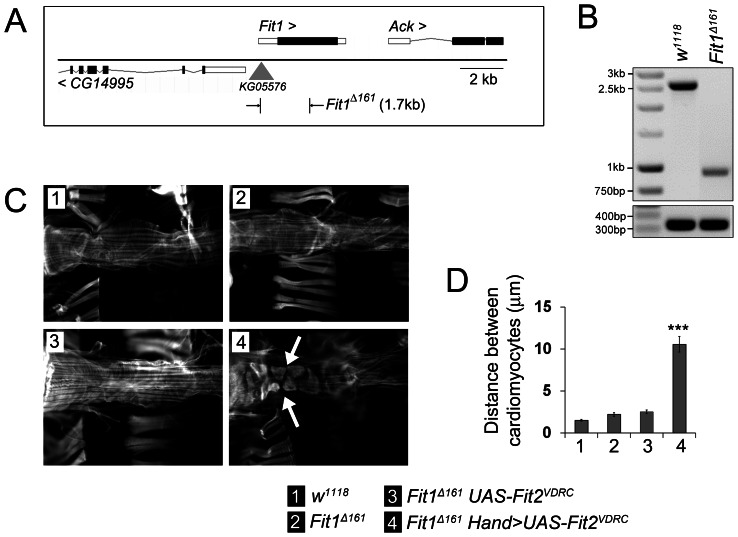
Cardiomyocyte *Fit2* compensates for the loss of *Fit1* to establish the cardiac syncytium. (**A**) Schematic showing the *Fit1* locus with the adjacent genes (*CG14995* and *Ack*) and the site of the P-element insertion used to create the 1.7 kb deletion mutant (*Fit1^Δ161^*). (**B**) PCR of genomic DNA from wild-type (*w^1118^*) and *Fit1^Δ161^* mutant flies. (**C**) Micrographs of adult hearts stained with phalloidin. The arrow highlights an abnormal cardiomyocyte phenotype. (**D**) Mean (±SEM) distance between neighbouring cardiomyocytes. *n* = 12–16 measurements from four to seven independent flies for each genotype. ***P<0.001 from all other genotypes. Legend applies to C & D.

The discrepancy between the phenotypes caused by the Fit1 VDRC and TRiP lines was likely due to the VDRC also targeting *Fit2*. This ‘double whammy’ would effectively knock-down both *Fit* genes and inhibit any functional compensation occurring between the two. To test this hypothesis, a *Fit1* mutant was generated via Δ2–3 mutagenesis. The P-element in *Fit1^KG05576^* allele was excised along with 1.7 kb of the *Fit1* gene in the 3′ direction to create the *Fit1^Δ161^* line (Figure 5A,B). *Fit1^Δ161^* flies were viable and had normal heart morphology (Figure 5). However, abnormal heart morphology was observed when *Fit2* was silenced (*Hand-Gal4; UAS.Fit2^VDRC^*) in *Fit1^Δ161^* mutant cardiomyocytes (Figure 5C), despite the same *Fit2* RNAi line, when used on its own, having no effect on heart function (compare Figs. 2 & 3 with Fig. 5C). These findings were confirmed when the distance between neighbouring cardiomyocytes was quantified (Figure 5D). Thus, loss of *Fit1* or reduced expression of *Fit2* is not sufficient to cause a heart phenotype, indicating that functional redundancy exists between *Fit1* and *Fit2*. Only when the expression of both genes is affected does a severe cardiomyopathy develop in *Drosophila*.

### Synchrony and contractility are disrupted when *Fit2* expression is silenced in *Fit1^Δ161^* mutant hearts

**Figure 6:**
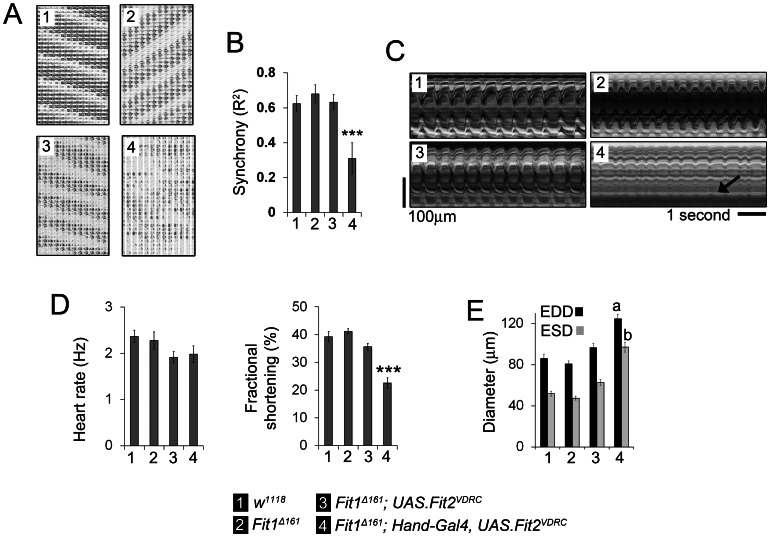
Cardiomyocyte *Fit2* compensates for loss of *Fit1* to maintain cardiac synchrony and contractility. (**A**) Representative cardiac actograms from four different genotypes. (**B**) Mean (±SEM) synchrony between different regions of the beating heart. *n* = 7 different flies for each genotype. ***P<0.01. (**C**) Representative M-modes of beating hearts showing the normal contractions in the *Fit1^Δ161^* mutant flies and shallow contractions in the *Fit1^Δ161^* mutant flies when *Fit2* is silenced (arrow). (**D**) Quantification of heart rate and fractional shortening in the four genotypes confirms that heart function was normal in the *Fit1^Δ161^* mutant flies and only becomes abnormal when *Fit2* expression was silenced in this mutant. (**E**) Quantification of end diastolic and systolic diameter (EDD and ESD, respectively). *n* = 10–11 flies per genotype, ***P<0.001; a = P<0.01 compared to all other genotypes' diastolic diameter; b = P<0.01 compared to all other genotypes' systolic diameter.

Synchronicity between discrete regions of the *Drosophila* heart was quantified and control (*w^1118^*) flies displayed regular synchronous cardiac contractility. *Fit1^Δ161^*, and *UAS.Fit2^VDRC^*; *Fit1^Δ161^* flies without *Hand-Gal4* displayed comparable synchronicity to *w^1118^* controls. In contrast, when *Fit2* was silenced (*Hand-Gal4; UAS.Fit2^VDRC^*) in *Fit1^Δ161^* mutant cardiomyocytes there was a significant loss of synchronicity between these regions of the heart (Figure 6A,B). There was no significant difference in heart rate between genotypes, whereas fractional shortening was significantly reduced in the *Hand-Gal4; UAS.Fit2^VDRC^ Fit1^Δ161^* mutants (Figure 6C,D). The *Hand-Gal4; UAS.Fit2^VDRC^ Fit1^Δ161^* mutants also had significantly increased end systolic and end diastolic diameters relative to the control and other genotypes (Figure 6E, P<0.01). Examples of the beating hearts and movement movies from the different genotypes can be seen in videos S9 to S16.

## Discussion

The current findings unequivocally demonstrate that cardiomyocytes must express members of the *Fermitin/Kindlin* family in order to develop as a functional syncytium. When *Drosophila* cardiomyocytes fail to couple together to form a cardiac syncytium, synchronous contractions and fractional shortening of the adult heart are significantly reduced, despite individual cardiomyocytes remaining myogenic. Studies of vertebrate hearts suggest a role for *Kind2* in cardiac development and function, however these studies are limited by the embryonic lethality of *Kind2* knock-out in the mouse model, and the lack of tissue specific *Kind2* silencing in the fish model [11], [12]. The aberrant phenotype caused by silencing orthologs of *Kind2* in the cardiomyocytes of an invertebrate demonstrates that the protein's role in cardiac development has been evolutionarily conserved and also reiterates the validity of using *Drosophila* to study genes relevant to mammalian cardiac physiology.

The aberrant heart phenotype caused by *Fermitin* knock-down in *Drosophila* reproduces many aspects of the cardiomyopathy caused by morpholino knock-down of *Kind2* in zebrafish [11], and supports the authors' conclusion that *Kind2* regulates heart development. Morpholino-knockdown in zebrafish disrupted angiogenesis but also affected intercalated disc formation in cardiomyocytes and significantly reduced fractional shortening. Previous studies in *Drosophila* have identified the *Fermitins* as mediators of muscle assembly [29], and the current findings extend this observation to include the assembly of a functional cardiac syncytium. Integrins and related adhesion proteins (ILK, Talin, Laminin A, Syndecan, Robo, and Slit) are necessary for heart assembly in *Drosophila* [30]–[32]. The current work is the first to demonstrate the functional consequences for the *Drosophila* heart when assembly of the cardiac syncytium is abnormal.

Human heart diseases attributed to intercalated disc dysfunction include arrhythmogenic right ventricular cardiomyopathy caused by mutations in D proteins (e.g. Desmoplakin, Plakophilin-2, and Desmoglein-2) [9], and hypertrophic and dilated cardiomyopathy caused by a missense mutation in the integrin-binding adhesion protein, Vinculin [10]. Integrins and related binding proteins (e.g. Vinculin and Talin) were also identified at intercalated discs in adult human cardiac tissue [33], [34]. Thus, there is a need to develop genetically tractable models with which to study the role of these proteins in cardiac development and function.

Intercalated disc-like structures have been identified between *Drosophila* cardiomyocytes, however there have been no empirical studies examining the molecular components nor the functional role of this structure in the heart [35]. The current data provide evidence that the β-integrin encoded by *myospheroid* is localised at the boundaries between adjacent cardiomyocytes, and also indicate that the development of a functional cardiac syncytium is strictly dependent on *myospheroid* as well as the expression of *Fermitins*. It would be informative to further assess the integrity of the cardiomyocyte junctions in both wild type and mutant flies using antibodies to cadherin and β-catenin [36]. The cardiomyopathy caused by silencing *Fit1* and *Fit2* phenocopied *myospheroid* knock-down and is therefore consistent with the *Fermitins* regulating β-integrin signalling and promoting cardiomyocyte coupling during development [31], [35]. There is evidence from mammalian models that Kindlins, via the FERM F3 subdomain, interact directly with the cytoplasmic tail of β-integrins [12], [18]. The FERM domain of *Drosophila* Fermitins is conserved from flies to humans and it is therefore speculated that Fermitins directly interact with the β-integrin *myospheroid* to form a signalling complex required for cardiomyocyte coupling. No study has yet manipulated the expression of mammalian *Kind2* solely in the cardiomyocytes, however it is predicted that a phenotype would develop that is similar to that seen in ventricular cardiomyocyte-targeted β1-integrin knockout mice [7]. Histological studies of the β1-integrin KO hearts revealed significant disruption of myofibrils and intercalated discs, with subsequent onset of dilated cardiomyopathy.

There was evidence that *Fit2* expression could compensate for *Fit1* in the *Drosophila* cardiomyocyte model. A *Fit1*-null mutant (*Fit1^Δ161^*) was generated that had normal heart morphology and function, indicating that Fit1 is not required for the development of a functional syncytium. This finding also suggested that *Fit2* might be compensating for the loss of *Fit1*. When *Fit2* was silenced in flies expressing *Fit1*, there was little impact on the heart. However, when the same *Fit2* RNAi line was expressed in the *Fit1*-null cardiomyocytes, it caused a severe cardiomyopathy. It can therefore be concluded that *Fit2* was responsible for syncytium development in the absence of *Fit1*.

In summary, the findings identify *Fermitins* as important mediators of cardiomyocyte coupling and heart function in *Drosophila*. Given the evolutionary conservation between invertebrate *Fermitins* and mammalian Kindlins, the evidence supports the hypothesis that disruption of cardiomyocyte *KIND2* may lead to abnormal intercalated discs and cardiomyopathies in humans.

## Supporting Information

Figure S1 **The Kindlin-2 FERM F3 subdomain, important for integrin-binding, is highly conserved in** ***Drosophila*** **Fermitins.**
(DOCX)

Figure S2 **The** ***Fit1^VDRC^*** **RNAi targets both** ***Fit1*** **and** ***Fit2*** **gene expression in the** ***Drosophila*** **heart.**
(DOCX)

Video S1 **Heart of** ***w^1118^*** **control.** Note: All videos show a representative 10 second sequence of the beating heart or movement movie derived from the beating heart. Videos S1 to S4, correspond to the data presented in Figure 3; videos S5 to S8 correspond to the data presented in Figure 4, and videos S9 to S16 correspond to the data in Figure 6.
(AVI)

Video S2 **Heart of** ***w^1118^*** **crossed with** ***UAS-Fit1^VDRC^*** **.**
(AVI)

Video S3 **Heart of** ***Hand-Gal4*** **;** ***UAS-Fit1^VDRC^*** **.**
(AVI)

Video S4 **Heart of** ***Hand-Gal4; UAS-Fit2^VDRC^*** **.**
(AVI)

Video S5 **Heart of** ***w^1118^*** **crossed with** ***UAS-Fit1^VDRC^*** **.**
(AVI)

Video S6 **Movement movie of** ***w^1118^ crossed with UAS-Fit1^VDRC^*** **heart.**
(AVI)

Video S7 **Heart of** ***Hand-Gal4; UAS-Fit1^VDRC^*** **.**
(AVI)

Video S8 **Movement movie of** ***Hand-Gal4; UAS-Fit1^VDRC^*** **heart.**
(AVI)

Video S9 **Heart of** ***w^1118^*** **.**
(AVI)

Video S10 **Movement movie of** ***w^1118^*** **heart.**
(AVI)

Video S11 **Heart of** ***Fit1^Δ161^*** **.**
(AVI)

Video S12 **Movement movie of** ***Fit1^Δ161^*** **heart.**
(AVI)

Video S13 **Heart of** ***Fit1^Δ161^, UAS-Fit2^VDRC^*** **.**
(AVI)

Video S14 **Movement movie of** ***Fit1^Δ161^*** **,** ***UAS-Fit2^VDRC^*** **heart.**
(AVI)

Video S15 **Heart of** ***Fit1^Δ161^, Hand-Gal4; UAS-Fit2^VDRC^*** **heart.**
(AVI)

Video S16 **Movement movie of** ***Fit1^Δ161^, Hand-Gal4; UAS-Fit2^VDRC^*** **heart.**
(AVI)
