# Supplementary material for: Fermitins, the Orthologs of Mammalian Kindlins, Regulate the Development of a Functional Cardiac Syncytium in Drosophila melanogaster
Source: PLoS One. 2013 May 15;8(5):e62958. doi: 10.1371/journal.pone.0062958 (PMC3655056; doi:10.1371/journal.pone.0062958)
Supplement: Figure S1 — The Kindlin-2 FERM F3 subdomain, important for integrin-binding, is highly conserved in Drosophila Fermitins. (DOCX) [file pone.0062958.s001.docx]

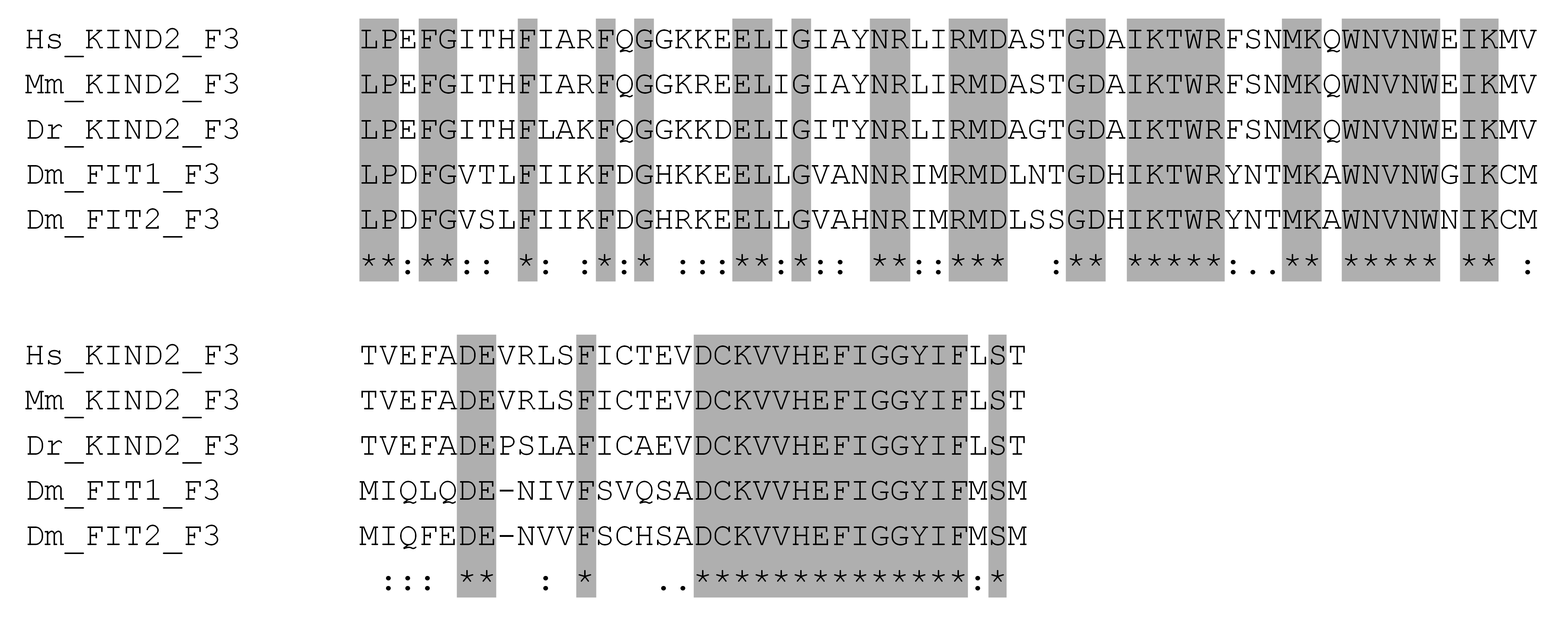


**Figure S1. The Kindlin-2 FERM F3 subdomain, important for integrin-binding, is highly conserved in *Drosophila* Fermitins.**

Clustal Omega alignments of human, mouse, and zebrafish KIND2 FERM F3 subdomain with *Drosophila* orthologs Fit1 and Fit2. The FERM F3 subdomain common to all three human Kindlin proteins, which is required for binding to the cytoplasmic tail of β-integrins, is conserved in *Drosophila* Fit1 and Fit2.
