# Supplementary material for: Fermitins, the Orthologs of Mammalian Kindlins, Regulate the Development of a Functional Cardiac Syncytium in Drosophila melanogaster
Source: PLoS One. 2013 May 15;8(5):e62958. doi: 10.1371/journal.pone.0062958 (PMC3655056; doi:10.1371/journal.pone.0062958)
Supplement: Figure S2 — The Fit1VDRC RNAi targets both Fit1 and Fit2 gene expression in the Drosophila heart. (DOCX) [file pone.0062958.s002.docx]

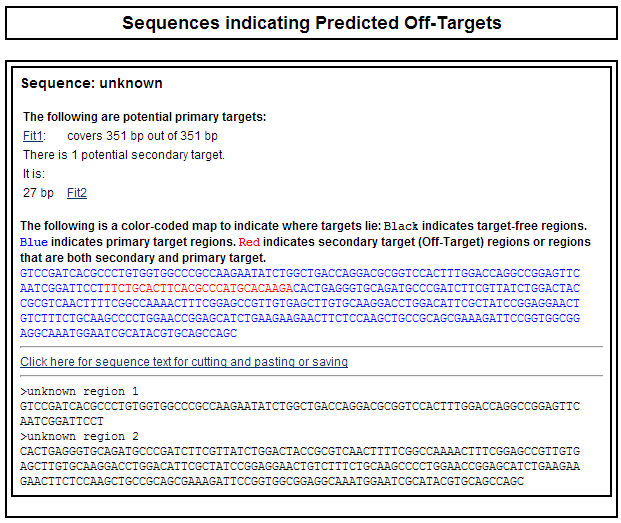


A


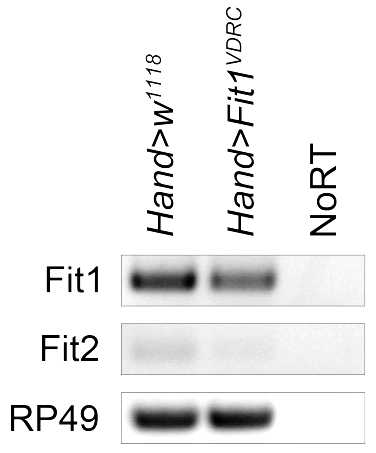


B

**Figure S2. The Fit1^VDRC^ RNAi targets both *Fit1* and *Fit2* gene expression in the *Drosophila* heart.**

(A) The Fit1^VDRC^ RNAi (construct ID 17050) is predicted to affect *Fit2* gene expression as well as *Fit1*. Off-target prediction was performed using the ‘Find OTEs’ program on the DRSC website (<http://www.flyrnai.org/RNAi_find_frag_free.html>). (B) Micrographs show *Fit1* and *Fit2* gene expression data from adult *Drosophila* heart tissue. Expression of both *Fit1* and *Fit2* was detected in cDNAs prepared from *Hand-Gal4; w^1118^* hearts (though *Fit2* expression was difficult to detect in the heart), whereas *Fit1* and *Fit2* were both affected in *Hand-Gal4; UAS.Fit1^VDRC^* flies. The housekeeping gene *RP49* was used as a positive control. NoRT – No Reverse transcriptase.
